# Effect of preparation design on marginal adaptation and fracture strength of ceramic occlusal veneers: A systematic review

**DOI:** 10.1002/cre2.653

**Published:** 2022-09-05

**Authors:** Samin Sirous, Arghavan Navadeh, Saeedeh Ebrahimgol, Faezeh Atri

**Affiliations:** ^1^ Departement of Prosthodontics, School of Dentistry Islamic Azad University (Khorasgan Branch) Isfahan Iran; ^2^ Private Practice Dubai UAE; ^3^ Department of Prosthodontics, School of Dentistry Tehran University of Medical Sciences Tehran Iran; ^4^ Department of Prosthodontics, Dental Research Center Dentistry Research Institute, Tehran University of Medical Sciences Tehran Iran

**Keywords:** fracture strength, marginal integrity, occlusal veneers, preparation design

## Abstract

**Objectives:**

This systematic review aims to investigate the effect of different preparation designs on the marginal fit and fracture strength of ceramic occlusal veneers.

**Materials and Methods:**

Based on the PICO question and the search terms, an electronic search was performed in Google Scholar, PubMed (MEDLINE), Scopus, Cochrane Library, Web of Science, Science Direct, Wiley, Ovid, and SAGE for articles published up to July 2022. After including English in vitro studies that evaluated posterior ceramic occlusal overlays at the posterior with ceramic restorations by following the PRISMA statement, the extracted data was tabulated. The methodological quality of the included studies was evaluated. Risk of bias assessment was done independently by two authors using the modified MINORS scale.

**Results:**

About 3138 search results were screened, of which 22 were selected due to their titles. Twenty‐one full‐text articles were assessed for eligibility. Seventeen in‐vitro studies were finalized for the extraction of quantitative data. All 17 articles had a low risk of bias and were retained. The influencing items for evaluating the research were different in most studies; therefore, qualitative synthesis of the results was feasible. They generally included preparation design, material thickness, depth of preparation in the tooth, internal divergence angle, and finish line. Meta‐analysis was not done due to heterogeneity of preparation types and evaluation methods. Results revealed that fracture resistance of occlusal veneers is higher than normal mastication force, and it is sufficient to prepare the occlusal surface, use a self‐etching primer for bonding, and an acceptable minimum ceramic thickness. The marginal discrepancy of occlusal veneers is clinically acceptable. However, this systematic review faces some limitations due to the lack of in vivo studies, different preparation designs in included studies, different follow‐ups, and lack of comprehensive explanations in articles.

**Conclusions:**

The preparation design of occlusal veneers influences both marginal adaptation and fracture resistance. Various preparation designs are proven to have clinically acceptable fracture strength and marginal adaptation.

## 1 INTRODUCTION

Preserving tooth structure is the principal goal of restorative dentistry, and by increasing the need for conservative and esthetic tooth restorations, the demand for partial‐coverage ceramics restorations is increasing (Blatz, 2002; Falahchai, Babaee Hemmati, Neshandar Asli, & Rezaei, 2020).

Besides caries as the main reason for tooth structure loss, some other non‐caries lesions, including erosion, abfraction, attraction, and fracture, lead to teeth's hard tissue breaking down and are needed to receive restorations (McCaul et al., 2004; Nascimento et al., 2011). Moreover, developmental tooth malformation may also need construction to reshape the tooth for biological, functional, esthetic, or social reasons (Bhaskar, 1986).

Tooth destruction predominantly affects the occlusal surface and functional cusps (Demarco et al., 2011), which can impact occlusal vertical dimension, esthetics, jawbone relationship, and occlusal stability (Attin et al., 2012; Mack, 1997). It is critical for the longevity of teeth and restorations to preserve remnant tooth structure (Van Dijken & Hasselrot, 2010).

The fracture strength of the dental material is one of the essential criteria to raise the survival rate of conservative restoration (Falahchai, Babaee Hemmati, Neshandar Asli, & Rezaei, 2020).

In addition, with the advance of new and reliable adhesive bonding techniques (Van Dijken & Hasselrot, 2010) and dental materials, selecting conservative treatment modalities, including less invasive restorations rather than aggressive treatments, is preferable.

Some less invasive alternative treatments such as conservative and esthetic partial coverage have been used currently, including inlays (no cuspal coverage), onlays (coverage of a minimum of one cusp), and overlays (all‐cusp coverage; Felden et al., 1998). Also, novel occlusal veneers with a non‐retentive design are used to restore the function and morphology of a defective occlusal surface (Johnson et al., 2014; Tsitrou & Van Noort, 2008).

To treat severe dental erosion, more conservative alternatives such as ultrafine restorations adhesively cemented have been preferred to traditional onlays or total crowns in posterior teeth (Johnson et al., 2014; Tsitrou & Van Noort, 2008), some studies have suggested occlusal veneers as they have high fracture resistance (Sasse et al., 2015). As many conventional preparation designs remove sound tooth structure, occlusal veneers with less invasive designs are increasingly sought‐after.

A study by Albelasy et al. (2021) has shown that material type and restoration thickness affect fracture resistance of CAD/CAM overlays. Moreover, some literature has determined occlusal ceramic fractures as the most common reason for restoration failure (Felden et al., 1998; Krämer & Frankenberger, 2005), which is affected by preparation design, bonding techniques, and thickness of the all‐ceramic restoration (Lima et al., 2013; Sasse et al., 2015).

Besides fracture resistance, marginal fitness is another main factor affecting long‐term restoration success (Suarez et al., 2003). A marginal gap results in the dissolution of luting cement and may cause restoration failure in the long run (Gu & Kern, 2003). Lack of marginal adaptation increases cement dissolution, leading to microleakage, secondary caries, periodontitis, marginal discoloration, and pulpal inflammation in some cases (Beuer et al., 2009; Sener‐Yamaner et al., 2017). Clinically, a marginal gap between 50 and 120 μm has been deemed acceptable (Heintze, 2007; Suarez et al., 2003).

Preparation design is an essential factor impacting fracture resistance in all‐ceramic restorations, such as rounding off all sharp angles (Krämer & Frankenberger, 2005). Nowadays, preparation designs for all‐ceramic restorations are modifications of conventional cast metal restorations (Stappert et al., 2008). Ultrathin one‐step with no‐prep polymer infiltrated ceramic network (PICN) overlays have indicated very high longevity and success (Ahmed et al., 2022). According to available data, the effects of preparation designs for an overlay are limited (Falahchai, Babaee Hemmati, Neshandar Asli, & Rezaei, 2020). Therefore, in this systematic review, the effect of the preparation design has been evaluated.

A systematic review by Vagropoulou et al. (2018) has shown that ceramic fractures were one of the restorations' complications, followed by retention loss and porcelain chipping; however, the survival rate for crowns was 95.38%, and for inlays and onlays, 90.89% and 93.50%, respectively, for 5 years, which are very high. However, this systematic review is aimed to investigate the effect of preparation design on the marginal fit and fracture strength of occlusal veneers.

## 2 MATERIALS AND METHODS

This systematic review followed the Preferred Reporting Items for Systematic Reviews and Meta‐Analyses (PRISMA) guidelines (McInnes et al., 2018). The focused PICO (population, intervention, control, outcome) question was whether specific preparation designs affect marginal adaptation and fracture strength of posterior occlusal ceramic veneers. The population, intervention, and outcome were defined as follows: the population was posterior occlusal veneers; the intervention was preparation design, and the outcome measures were marginal adaptation and fracture strength.

**Figure 1:**
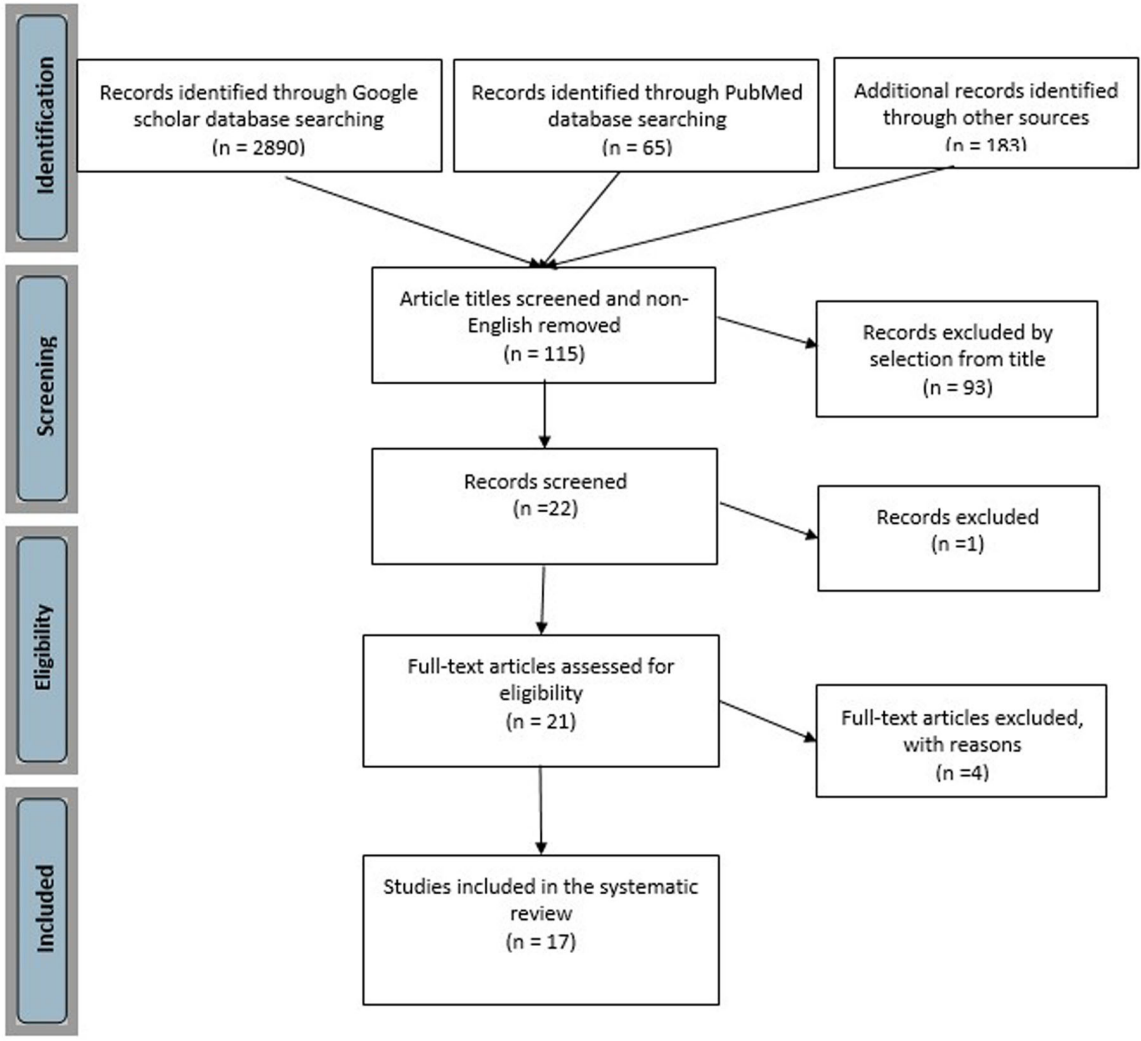
Decision flowchart of systematic literature review

An electronic literature search of articles until July 2022 was performed in Google Scholar, PubMed (MEDLINE), Scopus, Cochrane Library, Web of Science, Science Direct, Wiley, Ovid, and SAGE by using combinations of the following search terms: (preparation design OR tooth preparation OR dental preparation OR preparation type) AND (occlusal veneers OR overlay) AND (conventional ceramic restoration OR partial fixed dental prostheses OR inlay OR onlay OR partial crowns) AND (marginal adaptation OR marginal fit OR marginal gap OR marginal discrepancy OR marginal accuracy OR fracture resistance OR fracture strength OR bend strength OR fracture load OR fracture toughness OR flexural strength OR load to failure). Studies were selected by title and abstract according to the inclusion criteria. The eligibility criteria are listed in Table 1. Three independent reviewers (Arghavan Navadeh, Samin Sirous, and Saeedeh Ebrahimgol) initially screened the titles and abstracts of all potential matches, after which complete copies were retrieved and critically assessed according to the pre‐determined inclusion and exclusion criteria. The references of all selected articles were also manually searched for any other articles that may have been missed during the electronic search. Any disagreement regarding the eligibility of included studies was resolved by a fourth reviewer (Faezeh Atri). All relevant in vitro studies were included. (Figure 1) As all the included studies are in‐vitro, the statement on ethical approval, the “protection of human subjects and animals in research,” and informed consent are not indicated.

**Table 1:** Eligibility criteria

Inclusion criteria	Exclusion criteria
Studies evaluating occlusal overlays	Literature reviews
Studies in English	Composite restorations
In vitro studies	Crowns
Posterior restorations	Endocrowns
Ceramic restorations	Case reports, Finite element

Data were extracted from the included studies and tabulated with the following information: authors, groups, specimen type, sample size, restoration material, occlusal reduction, finish line, evaluation method, and conclusion (Table 2).

**Table 2:** Descriptive data of included studies

Authors and year of publication	Groups	Specimen type	Sample size	Restoration material	Occlusal reduction	Finish line	Evaluation method	Conclusion
Johnson et al. (2014)	1. 0.3 mm thickness MZ100 blocks 2. 0.6 mm thickness MZ100 3. 1.0 mm thickness MZ100 4. 0.3 mm thickness lava ultimate blocks 5. 0.6 mm thickness lava ultimate 6. 1 mm thickness Lava Ultimate	Maxillary molars	60	Paradigm MZ100 OR Lava Ultimate blocks	Occlusal thicknesses of 0.3, 0.6, and 1.0 mm		Universal testing machine	Restoration thickness had no statistically significant effect on fracture strength
Sasse et al. (2015)	1. Solely within enamel (EN) 2. Within enamel and dentin (E.D.) 3. Enamel and dentin with composite resin filling (E.C.) **subgroups (different thickness)** 0.3–0.6 mm 0.5–0.8 mm 0.7–1.0 mm	Intact molars	24 per group (in each group 3 subgroups (*n* = 8) with 3 different ceramic thickness (0.3–0.6 mm, 0.5–0.8 mm, 0.7–1 mm)	Lithium disilicate ceramic blocks	0.3–0.7 mm in the fissures + 0.6–1.0 mm at the cusps. An angle of 150 degrees was prepared between the cusps In group 3, the dentin core was reduced by 1.5 mm and a composite filling.		Universal testing machine	Only the group with 0.7 mm thickness in fissure, 1.0 mm at cusp survived cyclic loading without any damage. The occlusal ceramic veneers thickness had a statistically significant influence on fracture resistance. Suggestion: use a thickness of 0.7–1 mm for non‐retentive full‐coverage adhesively retained occlusal lithium disilicate ceramic restorations.
Albelasy et al. (2021)	1. e.max CAD 2. Vita enamic 3. Lava ultimate **Each group had Subgroups (Thickness)** 1. 1 mm 2. 1.5 mm	Intact maxillary molars	84	Lithium disilicate glass‐ceramic OR CAD/CAM resin composite OR polymer‐infiltrated ceramic	4 mm reduction		Thermal cycle/Kolmogorov–Smirnov and Shapiro–Wilk tests	All the CAD/CAM restorations in both thicknesses exhibited fracture resistance higher than normal and parafunctional masticatory forces.
Guess et al. (2013)	1. Palatal Onlay Standard 2. Palatal Onlay Thin 3. Palatal Onlay Ultrathin 4. Onlay Standard 5. Onlay Thin 6. Onlay Ultrathin 7. Complete Veneer Standard 8. Complete Veneer Thin 9. Complete Veneer Ultrathin	Intact human maxillary premolars	144 (9 groups of 16 specimens)	Pressable lithium disilicate glass‐ceramic	1. 2 mm palatal cusp reduction 2. 1 mm as above 3. 0.5 mm as above 4. 2 mm Buccal and palatal cusp reduction 5. 1 mm as above 6. 0.5 mm as above 7. Complete Veneer Standard: chamfer 0.8 mm Labial surface 8. Chamfer 0.6 mm Labial surface 9. Chamfer of 0.4 mm Labial surface	Chamfer	Cyclic mechanical loading and simultaneous thermocycling in a mouth‐motion simulator	No cracks or fracture failures during thermomechanical fatigue application. Group 7 showed higher mean fracture loads than palatal or occlusal onlays. Group 3 revealed higher fracture loads than complete veneers. Thin and ultrathin palatal and occlusal onlay in addition to complete veneer restorations failed predominantly because of extensive crack formation within the ceramic or cohesive fractures limited to the ceramic material.
Falahchai, Babaee Hemmati, Neshandar Asli, and Rezaei (2020)	1. (O) anatomical occlusal reduction 2. (O.S.) anatomical occlusal reduction with round shoulder 3. (O.G.) anatomical occlusal reduction with central groove 4. (OSG) anatomical occlusal reduction with round shoulder and central groove 5. (C) control group	Intact human maxillary first molars	50 (10 per group)	Zirconia‐reinforced lithium silicate	Pulpal depth of 2.5 mm and a width equal to half of the intercuspal distance. Buccal and lingual margins in groups O.S. and OSG received preparation with 1 mm width.	Groups O.S. and OSG received round shoulder	Universal testing machine	Group O showed significantly higher marginal adaptation than the group OSG, which had a more complex and more retentive preparation design. No significant difference in marginal adaptation of groups O, O.S., and O.G.
Falahchai, Babaee Hemmati, Neshandar Asli, and Neshandar Asli (2020)	1. Occlusal reduction(O) 2. Occlusal reduction with rounded shoulder (O.S.) 3. Occlusal reduction with a central groove (O.G.) 4. Occlusal reduction with rounded shoulder and central groove (OSG)	Intact human maxillary first molars	40 (4 groups of 10)	Zirconia‐reinforced lithium silicate (ZLS) ceramic	2.5 mm isthmus pulpal depth and a half of the inter‐cuspal distance width in the occlusal in groups 3 and 4. Occlusal prep for all cusps. 1 mm rounded shoulder prep in buccal and lingual margins in groups 2 and 4.	Rounded shoulder (for groups O.S. and OSG)	Video measuring machine for marginal gap measurement before and after cementation	Marginal gap size in group O was significantly smaller than that in group OSG. Gap size significantly increased in all groups after cementation.
Abu‐Izze et al. (2018)	1. Vita Suprinity with 0.5 mm (ZLS.5) 2. Vita Suprinity with 1 mm thickness (ZLS1) 3. In Vita Enamic with 0.5 mm (PIC.5) 4. In Vita Enamic with 1 mm thickness (PIC1).	Posterior teeth	60	Vita Zahnfabrik blocks	Simplified occlusal reduction corresponding to a lower second molar. Restorations were waxed in two occlusal thicknesses, 0.5 or 1.0 mm.		Kaplan Meier statistic of the number of cycles until failure. 5000 cycles at 200 N, followed by 450 N cycles. Axial load was carried out with a 4 Hz frequency in Biocycle V2 equipment (Biopdi)	ZLS.5 showed lower fatigue strength compared with PIC1, and PIC.5 and ZLS1 were similar. Restorations thickness increase reduced the concentrations of stresses on internal and external surfaces. Restorations thickness decreasing (less than 1 mm) has an impact on increasing its susceptibility to failure.
Ioannidis et al. (2019)	1. 0.5 mm zirconia 2. 1.0 mm zirconia 3. 0.5 mm lithium disilicate ceramic 4. 1.0 mm lithium disilicate ceramic 5. 0.5 mm PICN 6. 1.0 mm PICN 7. 0.5 mm resin composite 8. 1.0 mm resin composite	Human molars	80	Zirconia OR Lithium‐disilicate ceramic OR PICN OR Resin composite	Circular butt joint margins of 0.8–1.0 mm width+ 1.3–1.8 mm occlusal reduction + 3.0–4.0 mm minimal abutment height Two different thicknesses: 0.5 mm (range 0.3−0.7 mm) OR 1.0 mm (range 0.8−1.2 mm).		Universal testing machines thermo‐mechanically aged and then load applied	The differences of the load capacity bearing medians between the test and the control groups did not reach statistical significance for the 0.5 mm thick specimens. Within the 1.0 mm thickness groups, significant different medians were found.
Gupta et al. (2021)	1. Full crown (control) 2. Mesial‐occlusal‐distal‐facial onlay 3. Mesial‐occlusal‐distal‐lingual onlay 4. Overlay	Human premolars	32	Zirconia	G1. 1.5 mm depth cuts G2. 2 mm nonfunctional cusp reduction G3. 2 mm functional cusp reduction G4. 1 mm depth cuts	Rounded shoulder	thermomechanical loading with thermocycling/scanning electron microscope analysis	None of the specimens failed due to fracture or indicated any microcrack at 100× and 150×.
Emam and A Aleem (2020)	1. Lithium disilicate glass ceramics 2. Hybrid all‐ceramic material 3. Nano ceramic reinforced resin composite **Subgroups** (1). minimally occlusal veneer preparation (2). occlusal veneer preparation with marginal chamfer	Intact maxillary first premolars	60 (10 per subgroup)	Lithium disilicate glass ceramics OR Hybrid all‐ceramic material OR Nano ceramic reinforced resin composite	1 mm occlusal reduction, buccal and palatal margins approximately 5.0 mm from the CEJ	Chamfer	Vertical margin gap distance was measured by image analysis system For fracture resistance testing a computer‐controlled material testing machine	No statistically significant difference
Angerame et al. (2019)	1. Full‐coverage occlusal veneer 2. 1 mm cusp reduction with a marginal chamfer	Intact human maxillary molars	16 (2 groups of 8)	Lithium disilicate glass‐ceramic	1‐mm cusp reduction	G1. Rounded 90° shoulder G2. chamfer	Thermomechanical aging with a chewing simulator/scanning electron microscope analysis	No differences between the two groups in fracture load, fracture pattern, marginal quality, and internal fit.
Krummel et al. (2019)	1. Enamel Prep (EN) 2. Dentin/enamel Prep (ED) 3. Enamel/composite resin filling (E.C.) Prep **Each group had subgroups** 1. Self‐etching primer (S.E.) 2. Additionally etched enamel (E.E.)	Intact human molars	48 (3 groups of 16)	Lithium disilicate ceramic	Margins are in enamel in all Groups. 150 degrees angle between cusps. G1. 0.5 mm reduction G2. Prep extended into dentin G3. Prep extended into the dentin but the dentin core was 1.5 mm reduced and a composite filling		Computerized dynamic load testing with integrated thermocycling/universal testing machine	Significantly higher mean fracture resistance in bonding with selective enamel etching in comparison with self‐etching primer only to enamel and dentin. No difference in bonding solely to enamel or to enamel and composite.
Al‐Zordk et al. (2021)	1. Lithium disilicate 2. Zirconia 3. Polymer‐infiltrated ceramic **Each group had subgroups (bonding surface)** 1. Dentin 2. Dentin with intra‐coronal cavity 3. Dentin and composite filling	Intact mandibular first molars	90 (10 in each group)	lithium disilicate OR zirconia OR polymer‐infiltrated ceramic	150° angle between cusps. 2 mm cusp reduction. 1 mm pulpal depth, 2 mm bucco‐lingual width, 1.6 mm away from marginal ridge at intra‐coronal cavities	Tapered round‐end diamond was used	Thermo‐cycler machine and dynamic load aging/Universal testing machine	Different bonding had no significant effect on fracture resistance. All various bonds showed higher fracture resistance than normal masticatory forces in molars
Baldissara et al. (2019)	1. 0.5 mm Ceramic thickness 2. 0.8 mm Ceramic thickness 3. 1.2 mm Ceramic thickness	Intact human maxillary and mandibular molars	60 (3 groups of 20)	Lithium disilicate	Occlusal surfaces were sectioned using a rotary diamond blade		Ball mill machine as a simplified fatigue testing machine	Restoration thickness had significant effects on survival rate and fracture (thicker restorations showed higher survival rate and less fracture).
Huang et al. (2020)	1. Occlusal surface coverage (O) 2. Occlusal and lingual surface coverage (OF) 3. Occlusal, lingual, and mesial surface coverage (POF) 4. Occlusal, lingual, mesial, and distal surface coverage (POFP) 5. Full crown	Intact human maxillary premolars	40 (8 per group)	Lithium disilicate glass ceramic	1. 0.7–1.0 mm occlusal 2. Same as group 1 + 0.5–1.0 mm lingual 3. Same as group 2 + 0.5–1.0 mm mesial 4. Same as group 3 + 0.5–1.0 mm distal 5. Same as group 4 + 0.5–1.0 mm buccal	0.5‐mm‐thick marginal chamfer	Universal testing machine	As the number of restored axial walls increased, the fracture resistance decreased, and the maximum principal stress in the restoration increased. Minimally invasive preparation is recommended.
De Jesus Tavarez et al. (2014)	1. Control, healthy unrestored teeth 2. Teeth restored with ceramic fragments 3. Teeth restored with ceramic overlays	Intact maxillary premolars	30 (10 per group)	Feldspathic ceramic	1. No prep 2. Unprepared restored with 1 mm ceramic 3. Extracoronal prep restored with 1 mm overlays 2 mm occlusal box, one third of the intercuspal distance, 1.5 mm proximal box, and a 2 mm gingival wall from the pulpal wall		Universal testing machine	The teeth in G2 and G3 presented with type II fractures that involved a small portion of the coronal tooth structure and cohesive failure. G1 presented with more extensive fractures involving the root. G2 may offer greater resistance to fractures compared to G3.
Stappert et al. (2005)	1. Control group 2. MOD inlay 3. Palatal partial coverage 4. Palatal and buccal partial coverage 5. Complete veneer	Intact human maxillary premolars	80 (5 groups of 16 specimens)	Pressed ceramic	G1. No prep G2. 3 mm deep occlusal box and a 2 mm isthmus. The proximal boxes ended 1 mm short of CEJ G3. 2 mm palatal cusps reduction and following basic prep G4. 2 mm buccal cusps reduction G5. Basic prep and 0.8 mm facial chamfer	Chamfer	Computer‐controlled masticatory simulation and fracture load testing	All specimens survived dynamic loading. The fracture load values of the unprepared teeth and those prepared for inlays and complete veneers were significantly higher than those of which cusps had been reduced in preparation for a partial coverage restoration.

### 2.1 Quality assessment

Each included study was evaluated by three reviewers (Arghavan Navadeh, Samin Sirous, and Saeedeh Ebrahimgol) independently for the methodological quality, according to the following criteria: clearly stated aim, contemporary groups, clearly stated preparation method, baseline similarity of groups, randomization of specimens, clearly stated evaluation method (marginal gap/strength), blinding of the examiner, sample size calculation and power analysis, and adequate statistical analyses. The items were scored on a scale of 0–2; 0 if not reported; 1 when reported but inadequate; and 2 when reported and adequate. The overall score was considered from 18 for each study, and the articles were classified as high (0–6), medium (7–12), and low risk (13–18) of bias based on their score (Table 3).

**Table 3:** Authors' judgments about risk of bias for each included study

Authors and year of publication	Clearly stated aim	Contemporary groups	Baseline similarity of groups	Randomization of specimens	Clearly stated preparation method	Clearly stated evaluation method (marginal gap/strength)	Blinding of the examiner	Sample size calculation and power analysis	Adequate statistical analyses	Total score	Risk of bias
Johnson et al. (2014)	2	2	2	2	1	2	2	2	2	17	Low
Sasse et al. (2015)	2	2	2	2	2	2	2	2	2	18	Low
Albelasy et al. (2021)	2	2	2	2	1	2	2	2	2	17	Low
Guess et al. (2013)	2	2	2	2	2	2	2	2	2	18	Low
Falahchai, Babaee Hemmati, Neshandar Asli, and Rezaei (2020)	2	2	2	2	2	2	2	2	2	18	Low
Falahchai, Babaee Hemmati, Neshandar Asli, and Neshandar Asli (2020)	2	2	2	2	1	2	2	2	2	17	Low
Abu‐Izze et al. (2018)	2	2	1	2	1	2	2	2	2	16	Low
Ioannidis, et al. (2019)	2	2	2	2	1	2	2	2	2	17	Low
Gupta et al. (2021)	2	2	2	2	1	2	2	2	2	17	Low
Emam and A Aleem (2020)	2	2	2	2	2	2	2	2	2	18	Low
Angerame et al. (2019)	2	2	2	2	2	2	2	2	2	18	Low
Krummel et al. (2019)	2	2	2	2	2	2	2	2	2	17	Low
Al‐Zordk et al. (2021)	2	2	2	2	1	2	2	2	2	17	Low
Baldissara et al., (2019)	2	2	2	2	1	2	2	2	2	17	Low
Huang et al. (2020)	2	2	2	2	2	2	2	2	2	18	Low
De Jesus Tavarez et al. (2014)	2	2	2	2	1	2	2	2	2	17	Low
Stappert, et al. (2005)	2	2	2	2	2	2	2	2	2	18	Low

*Note*: The items are scored as follows: 0, not reported; 1, reported but not adequately; or 2, reported adequately.

## 3 RESULTS

### 3.1 Identification of studies

A total of 3138 search results were screened. By evaluating the abstracts, 22 articles were selected. Of the 22 articles, one study was eliminated due to lack of data; another one was excluded since the full text could not be retrieved, and two case reports and one finite element were eliminated. Therefore, 17 articles were selected for extracting the quantitative data. The results of data extraction for each study are presented in Table 2.

As there was no specific scale to assess study risk or quality, in this systematic review, two authors (Samin Sirous/Faezeh Atri) independently evaluated the risk of bias by using the modified MINORS scale, which is a methodological index for non‐randomized studies (Slim et al., 2003). Each item was scored between 0 and 2; 0 for not reported items; 1 when reported but inadequate; and 2 when both reported and adequate. 18 was the ideal score; if the study score was between 13 and 18, the authors determined it as low risk; 7–12 fell under medium risk, and scores lower than 6 were counted as high risk. As all the studies showed a low risk of bias, they were retained in the review (Table 3).

The included studies were in vitro. The specimens of studies varied from maxillary premolars (Guess et al., 2013), maxillary molars (Falahchai, Babaee Hemmati, Neshandar Asli, & Rezaei, 2020), or maxillary first molars (Falahchai, Babaee Hemmati, Neshandar Asli, & Neshandar Asli, 2020), and so on. However, in the study by Abu‐Izze et al. (2018), wherein specimens are simply described as posterior teeth, or in the study by Ioannidis et al. (2019), the tested teeth were mentioned as human molars (Table 2).

Falahchai, Babaee Hemmati, Neshandar Asli, and Rezaei (2020) and Falahchai, Babaee Hemmati, Neshandar Asli, and Neshandar Asli (2020) evaluated the marginal gap in the specimens, and Gupta et al. (2021), Emam and A Aleem (2020), and Angerame et al. (2019) assessed both marginal adaptation and fracture resistance of the specimens, and it is noteworthy that other articles studied fracture strength.

The influencing items for evaluating the research were different in most of the studies. In general, they are as follows: preparation design, material thickness, depth of preparation in the tooth (cavity depth), internal divergence angle, and finish line.

Meta‐analysis could not be calculated because of the heterogeneity of preparation types and evaluation methods.

Qualitative analysis of included studies came to the following results. The marginal discrepancy and fracture resistance of occlusal veneers of all preparation designs are clinically acceptable. The strength of occlusal veneers is higher than normal mastication force (Johnson et al., 2014), and it is sufficient to prepare only the occlusal surface, use a self‐etching primer for bonding, an acceptable minimum ceramic thickness, and as a conclusion, there is no need for extensive reduction.

## 4 DISCUSSION

This systematic review aimed to evaluate the effect of different aspects of preparation designs of occlusal veneers on marginal adaptation and fracture resistance. The analysis of related articles determined that different aspects could influence marginal adaptation and fracture strength. Since overlays are considered a less invasive technique in some cases for reconstructing teeth for mastication and esthetics, it is essential to evaluate which modification increases fracture strength and reduces the marginal gap. In the following section, we discussed related articles that present preparation modifications, including different dental bonding surfaces, number of axial surfaces, different occlusal thickness, type of finish line, and preparation design.

### 4.1 Different bonding surface (enamel or dentin)

The success of an occlusal overlay is affected by different factors, with bonding technique being one of them. The type of bonding and the type of dental surfaces involved influence the bond strength. Due to various conditions, there are different indications for each bonding technique. Sasse et al. (2015) reported that luting to dentin or composite yields significantly higher fracture strength than luting to enamel only. This is due to different bonding techniques, namely the use of self‐etching primer that increases dentin bond strength as opposed to the total‐etch technique, which is used in several other studies. However, another study by Krummel et al. (2019) proved that additional etching of enamel or total‐etch, that is, selective enamel etching increases fracture strength when compared to self‐etching primer when bonding to dentin and enamel. They proposed that the recent study's higher fracture strength in dentin could be due to the improvement of a newly developed so‐called universal bonding system. On the other hand, the study by Walid Al‐Zordk et al. (2021) has shown that the fracture resistance within veneers bonded to dentin, dentin with intra‐coronal cavity, and dentin with the composite filling was not significantly different. However, their fracture resistance is above human regular masticatory forces in molars. Also, by varying dental bonding surfaces, different overlays' materials have to be considered (Al‐Zordk et al., 2021).

### 4.2 Different thicknesses of cavity preparation

It is determined that overlays with various occlusal thicknesses could tolerate loads higher than human masticatory forces ranging from 585 to 880 N (Johnson et al., 2014). Although an increase in the occlusal thickness of overlay restorations can lead to higher fracture resistance, further reduction more than carious lesion or fracture to increase the occlusal thickness of the overlays is not recommended. In Albelasy's study, the thickness of both 1 and 1.5 mm occlusal veneers exhibited fracture resistance higher than functional and parafunctional masticatory forces (Albelasy et al., 2021). By evaluating the influence of restoration thickness on fracture resistance, Sasse et al. (2015) concluded that occlusal ceramic veneer thickness affects fracture resistance. They suggested a minimum thickness of 0.7–1 mm when using a self‐etching primer which can increase fracture resistance compared to thinner restorations. In another study by Abu‐Izze et al. (2018), 1‐mm hybrid ceramics achieved better results in the fatigue test compared with 0.5‐mm Zirconium‐reinforced lithium silicate restorations, the rise in the thickness of the restorations significantly decreased the concentrations of stresses on different internal and external surfaces, and the stress concentration of Zirconium‐reinforced lithium silicate at the adhesive interface was higher in comparison to hybrid ceramics. In Baldissara's study (Al‐Zordk et al., 2021), comparing different groups, 0.8 mm may be considered an acceptable compromise between fracture resistance and tooth reduction as a threshold value.

Also, Ioannidis et al. (2019) indicated both 0.5 and 1.0 mm thicknesses as suitable for minimally invasive lithium‐disilicate ceramic (IPS e.max Press), zirconia (Vita YZ H.T.), PICN (Vita Enamic), and resin composite (Lava ultimate) overlay restorations in the posterior area. In Guess et al.'s (2013) in vitro study, ceramic thickness did not influence the fracture strength of occlusal veneer restorations. As a result, 1‐ and 0.5‐mm preparation depth reduction did not impair fracture resistance of pressable lithium‐disilicate ceramic onlays. This might be because this study was conducted on premolar specimens rather than molars used in the other studies. In Johnson et al.'s (2014) study with Paradigm MZ100 or Lava Ultimate, where blocks were fabricated at minimal occlusal thicknesses of 0.3, 0.6, and 1.0 mm, the restoration thickness had no statistically significant effect on fracture resistance. This can probably be attributed to the fact that these materials contain composite, which has better survival under cyclic loading than conventional ceramic.

### 4.3 Number of involved axial surfaces

In terms of some preparation surfaces, overlays reveal a higher fracture resistance than normal mastication force. In most cases, adding axial surfaces to the preparation does not significantly improve fracture resistance. However, some full‐crown cases show higher load resistance than overlays. Huang et al. (2020) concluded that veneers with only occlusal surface coverage have higher fracture strength than those covering three or more surfaces; on the other hand, occlusal‐only and occlusal plus lingual surface coverage veneers exhibited higher fracture strength than full crowns. Conversely, Guess et al. (2013) revealed the highest load values in complete veneer preparations that included both the palatal and buccal premolar cusps and the labial surface, and in 2014 a study indicated that overlays have lower fracture strength than ceramic fragments restorations (De Jesus Tavarez et al., 2014). In 2005, Stappert et al. (2005) studied the effect of varying preparation designs on reliability and fracture resistance of partial coverage premolar restorations and concluded that complete veneers, including the facial and masticatory surfaces (buccal and palatal cusps), showed comparable fracture resistance to that of unprepared natural teeth and are, therefore, superior to veneers covering only the buccal and/or palatal cusps. In the study by Gupta et al. (2021) with 3Y‐zirconia restorations with strict bonding techniques, full or partial coverage are viable restorations. However, this study suggested to avoid occlusal contact on restorations margins.

### 4.4 Different preparation design and finish line (marginal gap)

The reviewed studies also proved a relationship between finish line design and marginal adaptation. Falahchai, Babaee Hemmati, Neshandar Asli, and Rezaei (2020) showed that overlays prepared most conservatively with an anatomical occlusal reduction yielded the highest fracture resistance compared with those that included a rounded shoulder preparation or/and a central groove. In another study, Falahchai, Babaee Hemmati, Neshandar Asli, and Neshandar Asli (2020) proved that more invasive preparation designs are not necessary as the marginal adaptation of veneers prepared with only an anatomical occlusal reduction showed minimum marginal gap as opposed to those including a rounded shoulder or/and central groove. In Angerame et al. (2019), teeth were prepared either with a rounded 90‐degree shoulder finish line or a minimally invasive chamfer, and both results were similarly satisfactory. Emam and A Aleem (2020) tested minimally invasive occlusal veneer preparations resembling occlusal erosion against occlusal veneer preparations with a marginal chamfer and found no statistically significant difference between marginal gap distances.

The above data indicates that the preparation of overlays influences the fracture strength and marginal integrity of restorations. However, a systematic review mainly including in vitro studies cannot give a high level of evidence since its results cannot be extrapolated to humans. Also, this review had limitations due to using different teeth (premolars vs. molars), differently used materials, measuring techniques, and location of the teeth in included studies, and lack of comprehensive explanations in articles. Quantitative data analyses are prevented due to the high heterogeneity of the included articles. Some parameters have been considered based on a few studies or even one, and various other factors are not evaluated in studies; therefore, results should be drawn cautiously, and further studies and especially clinical trials with long periods of follow‐up, are required to assess different modifications of overlay preparation design.

## 5 CONCLUSION

Within the limitations of this systematic review, and according to the findings, the following conclusions were drawn:

1. The preparation design of overlays influences both marginal adaptation and fracture resistance.
2. Various preparation designs can be used with occlusal veneers to safely restore posterior teeth as these restorations are proven to have clinically acceptable fracture strength and marginal adaptation.
3. Fracture resistance of occlusal veneers is higher than the normal range of human masticatory force; therefore, aggressive preparation to increase the fracture resistance is not recommended.

## AUTHOR CONTRIBUTIONS

Faezeh Atri proposed the study concept. Arghavan Navadeh, Samin Sirous, and Saeedeh Ebrahimgol searched and gathered the relevant articles and analyzed them, and then Faezeh Atri approved the final ones. All the authors contributed to article evaluation and manuscript writing. The final manuscript is approved by all authors.

## CONFLICT OF INTEREST

The authors declare no conflict of interest.

## Data Availability

The data that support the findings of this study are available from the corresponding author upon reasonable request.
